# Cow Dung-Based Biochar Materials Prepared via Mixed Base and Its Application in the Removal of Organic Pollutants

**DOI:** 10.3390/ijms231710094

**Published:** 2022-09-03

**Authors:** Xiaoxin Chen, Gengxin Yu, Yuanhui Chen, Shanshan Tang, Yingjie Su

**Affiliations:** 1 College of Life Sciences, Jilin Agricultural University, Changchun 130118, China; 2 Key Laboratory of Straw Comprehensive Utilization and Black Soil Conservation, Ministry of Education, Jilin Agricultural University, Changchun 130118, China

**Keywords:** industrial wastewater, organic pollutants, cow dung, biochar materials, adsorption

## Abstract

Cow dung (CD) is a waste product of livestock production. Improper disposal of a large amount of CD will cause environmental pollution. In this work, three biochar materials based on CD (BMCD) were prepared by using three types of base, including KOH, NaOH, and mixed base (MB, a mixture of equal mass NaOH and KOH) as activators to investigate the different physicochemical properties of BMCDs (BMCD-K, BMCD-Na, and BMCD-MB). The objective was to verify the effectiveness of MB activation in the preparation of biochar materials. The results show that MB has an effect on the structural characteristics of BMCDs. In particular, the surface area and total pore volume, the specific surface area, and the total pore volume of BMCD-MB (4081.1 m^2^ g^−1^ and 3.0118 cm^3^ g^−1^) are significantly larger than those of BMCD-K (1784.6 m^2^ g^−1^ and 1.1142 cm^3^ g^−1^) and BMCD-Na (1446.1 m^2^ g^−1^ and 1.0788 cm^3^ g^−1^). While synthetic dye rhodamine B (RhB) and antibiotic tetracycline hydrochloride (TH) were selected as organic pollutant models to explore the adsorption performances, the maximum adsorption capacities of BMCD-K, BMCD-NA and BMCD-MB were 951, 770, and 1241 mg g^−1^ for RhB, 975, 1051, and 1105 mg g^−1^ for TH, respectively, which were higher than those of most adsorbents. This study demonstrated that MB can be used as an effective activator for the preparation of biochar materials with enhanced performance.

## 1. Introduction

Industrial wastewater is one of the most acute causes affecting water safety [1]. Because it causes significant pollution, it not only threatens plants, animals and environment, but also poses a great danger to human health, so it has gradually become one a focal point for researchers [2,3,4]. Given this serious problem, various methods have been developed for the removal of organic pollutants (like antibiotics and dyes) from water, such as physical treatment including adsorption and reverse osmosis [5,6], and chemical treatment (using chemical reagents) including degradation and precipitation [7,8]. Among these methods, adsorption is widely used for wastewater treatment because of its environmental friendliness, low cost, and simple operation [5,9]. Normally, adsorbent is one of the most important factors affecting the effect of adsorption treatment and adsorption process. Biochar materials have found wide applications as adsorbents for the treatment of polluted wastewater due to their unique advantages, like high pore structure and large surface area [10,11]. To date, it is still necessary to explore new carbon resource of biochar materials in order to better cope with wastewater pollution treatment.

Cow dung (CD), a waste product of livestock production, is generally considered to be the undigested food residue of grazing animals (cattle) [12,13]. As cattle farming and cattle-feeding operations increase, so does the yield of the hard-to-handle waste CD. Since these waste emissions demonstrate a high risk of pathogen transmission, their disposal is considered a major challenge for the cattle and livestock industry [14,15]. In the past decades, reports on comprehensive utilization of CD have emerged one after another. Because CD contains a large amount of undigested lignocellulose and inorganic salts, it is often used to produce bio-fertilizers, solid fuel, and friction composites, etc. [16,17]. With the progress of science and technology, only solving the problem of using waste CD is not enough to meet the development of society. Therefore, more and more researchers focus on how to use CD to produce high-performance products, to further improve its added value and release its economic potential. Recently, it has been demonstrated that CD-based biochar materials represented good porous structure and ideal surface area [18,19,20]. Obviously, this provides a new idea for many researchers, but how to further improve the performance of CD-based biochar materials remains a challenge.

Like many biochar materials, the biochar materials prepared by only carbonization have a low surface area and poor porosity, so further activation is required to improve their properties [21,22,23,24]. The selection and the number of different activators have significant influence on the porosity of the materials. Due to the different behaviors during activation, sodium hydroxide and potassium hydroxide are often used as activators for biochar materials. Over the past three years, our team constituted mixed base (MB) from equal masses of sodium hydroxide and potassium hydroxide, and have also been exploring the influence of MB on the preparation of biochar materials. Our previous studies have shown that the activation effect of MB on some biomass materials (such as corn straw, fungal hyphae, and edible fungus substrate) [21,22,23,24] may be better than that of the single base. However, there are few reports on whether the effect of preparing CD-based biochar materials with MB as activator is better than that of single base activation, which provides great motivation for us to explore this topic.

In this work, CD was used as raw material, after carbonization, three base including sodium hydroxide, potassium hydroxide, and MB were used as different activators for preparing CD-based biochar materials. The focus of this study is to explore the possibility of preparing CD-based biochar materials with enhanced performance via MB and the influence of different activators on the properties of biochar materials. Different characterization methods have been used to test the CD-based biochar materials in order to study the differences of physical and chemical structures of biochar materials with different activators. In addition, tetracycline hydrochloride (TH) and rhodamine B (RhB) were selected as organic pollutant models to explore the effects of different activators on the adsorption performances of CD-based biochar materials. This study aims to prepare a series of CD-based biochar materials with better performance. More importantly, this simple and efficient method can be further applied to produce other biochar materials.

## 2. Results and Discussion

### 2.1. Characterization Results

The change of CD with temperature under nitrogen protection was measured by TGA and DTG curves, and the results are shown in Figure S1. It can be seen that, with the increase of temperature, there are two obvious pyrolysis stages of CD. When the temperature is below 275 °C, the weight of the sample decreases slowly, which may be due to the evaporation of the water of crystal remaining in the sample as the temperature increases [25]. As the temperature continued to rise to 500 °C, the samples showed a dramatic weight loss, which could be explained by the high temperature decomposition of the oxygenated components of CD (fat, protein, and lignocellulose, etc.) into tar and combustible gases [26]. However, when the temperature exceeds 500 °C, the curve becomes stable without further weight loss, indicating that the carbonization process is complete. The trend of DTG curve was consistent with that of TGA curve, thus, the carbonization temperature was chosen as 500 °C.

**Figure 1:**
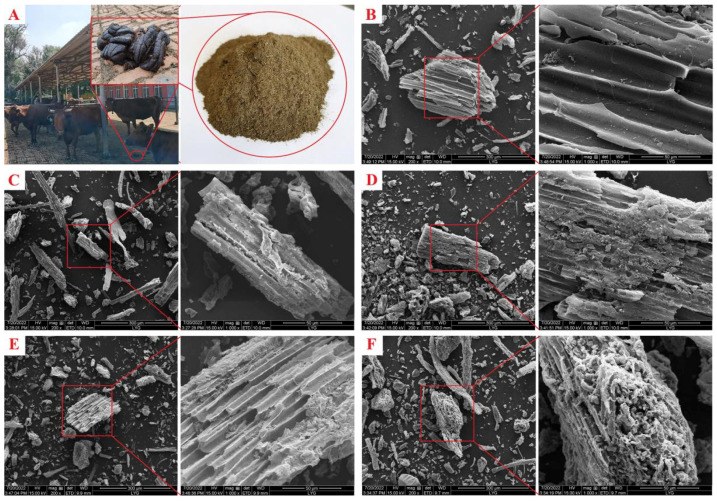
Digital photo of (**A**) CD. SEM images of (**B**) CD, (**C**) CDC, (**D**) BMCD-K, (**E**) BMCD-Na, and (**F**) BMCD-MB.

The main component of CD is lignocellulose of straw or forage, and the results of SEM can show clear and obvious fiber structure (Figure 1B). With the completion of the carbonization process, the sample was dehydrated significantly (CDC), and the originally smooth fiber structure became wrinkled and broken (Figure 1C). Figure 1D–F shows the morphological observation results of BMCDs prepared by different activators. It can be seen that all of them have drastic activation and fragmentation phenomenon, and the degree of fragmentation is different due to different activators. In particular, BMCD-MB is more severe. This is related to the activation efficiency of different activators, and also indicates the effectiveness of MB activation compared with single base under the same conditions.

**Figure 2:**
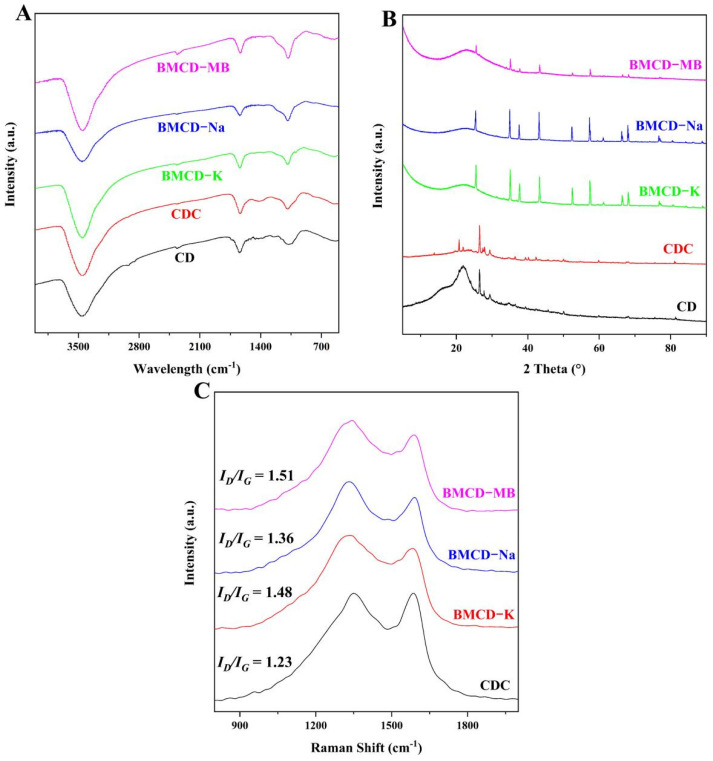
(**A**) FT-IR spectra, (**B**) XRD pattern, and (**C**) Raman spectra of biochar materials.

FT-IR were used to investigate the functional groups of the materials, and the results were shown in Figure 2A. The broadband corresponding to the -OH stretching vibration of hydroxyl functional groups in the range of 3450–3410 cm^−1^ is still existed, showing the existence of residual oxygen-containing functional groups on the resultant biochar materials [25,26]. The CDC and BMCDs had weaker -CH bonds (2910–2930 cm^−1^, -CH group’s symmetric and asymmetric stretching vibration peaks) than the CD, indicating that a considerable quantity of hydrogen was removed during the carbonization and activation procedures [27]. The aromatic ring’s C=C stretching is indicated by the peak at 1619 cm^−1^. This can be explained by the fact that the C-H bond may have decomposed into the more stable aromatic C=C bond after high-temperature preparation. This may be due to the conjugation of the C=O group to the aromatic ring and the formation of the carbonyl-containing group, which suggests that carbonyl-containing groups were formed during the aromatization process [28]. In addition, the overlapping bands in the 1000–1300 cm^−1^ region correspond to the stretching vibrations of the C-O and C-N heterocycles [27,28]. The characteristic peaks below 1100 cm^−1^ are mainly generated by the C-C skeleton vibrations, the bending vibrations of oxygen-containing groups (-CH, O-H), and the stretching vibrations of some single bonds (C-O, C-N) [28]. The presence of these large number of functional groups is likely to enhance the performance of BMCDs as adsorbents.

XRD were used to investigate the crystallinity in the carbons, and the results were shown as Figure 2B. The appearance peaks near 17° and 23° are due to the presence of a large amount of cellulose in the CD [29]. After carbonization (CDC) and activation (BMCDs), BMCDs still exhibited a diffraction peak within the range of 10°–30°. This may be assigned to the plane of graphite structure (002), indicating that BMCDs had a classic local-order structure of carbon materials [9,11]. The intensity of this peak was weak and it was a broad peak, which can be explained by the fact that BMCDs contained both crystalline and amorphous structures [9,11]. Like many dung-based biochars [18,19], there are many irregular but distinct peaks, which can be explained by the inorganic salts (e.g., sodium chloride), bone meal, and shell meal, which were fed in the process of animal husbandry, connected to the carbon matrix during high temperature pyrolysis. However, there is no doubt that BMCDs were not crystalline but amorphous.

Raman tests were used to investigate the presence of defects in the biochar materials, the results were shown as Figure 2C. There were two typical peaks, the peak around 1330 ± 25 cm^−1^ is D-band associated with amorphous carbon and the G-band around 1550 ± 30 cm^−1^ is associated with graphitic carbon [30]. The intensity ratio of D-band (*I_D_*) and G-band (*I_G_*) (*I_D_/I_G_*) is an important parameter to measure the degree of defect and disorder in carbons. The *I_D_/I_G_* values of BMCD-K, BMCD-Na, and BMCD-MB were 1.48, 1.36, and 1.51. Compared with the *I_D_/I_G_* values of CDC (1.23), the increase in BMCDs indicate the presence of more amorphous carbon structures generated in the biochar materials after activation [31].

**Figure 3:**
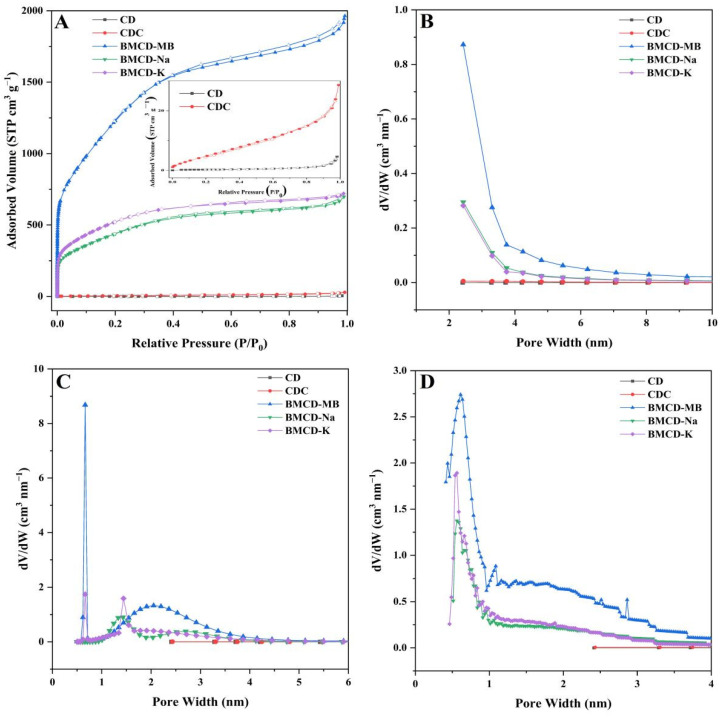
(**A**) N_2_ adsorption-desorption isotherms of BMCD-K, BMCD-Na, and BMCD-MB (inset: N_2_ adsorption-desorption isotherms of CD and CDC. Pore size distribution curves of CD, CDC, BMCD-K, BMCD-Na, and BMCD-MB based on (**B**) BJH, (**C**) NLDFT, and (**D**) H-K methods.

N_2_ adsorption-desorption isotherms were used to obtain the specific surface areas and porosity of the biochar materials, the results were shown as Figure 3 and Table 1. As can be seen from the inset of Figure 3A, the specific surface area and the total pore volume of CDC (23.8 m^2^ g^−1^, 0.0436 cm^3^ g^−1^) have been increased relative to that of CD (0.9 m^2^ g^−1^, 0.0071 cm^3^ g^−1^) after carbonization, which may be caused by high-temperature pyrolysis [25,26]. It is worth noting that CDC is not sufficient to support the excellent performance of CD-based on these results; thus, further activation is required to improve the specific surface area and pore volume.

After activation, the porosity of BMCDs were greatly improved. The specific surface areas of BMCD-K, BMCD-Na, and BMCD-MB were 1784.6, 1446.1, and 4081.1 m^2^ g^−1^. The total pore volume of BMCD-K, BMCD-Na, and BMCD-MB were 1.1142, 1.0788, and 3.0118 cm^3^ g^−1^. All BMCDs displayed type I isotherms, showing the existence of a significant amount of micropores within the carbon compounds [29,31]. The volume of micropores of BMCD-K, BMCD-Na, and BMCD-MB were 1.0239, 0.9124, and 2.5583 cm^3^ g^−1^, and they occupy 91.90%, 84.56%, and 84.94% of the total pore volume, respectively. Meanwhile, type IV hysteresis loops were discovered in all the samples’ isotherms, although they were quite narrow, indicating that all of the samples included a modest quantity of mesopores [21,22]. According to the results of the pore distributions (Figure 3B–D), BMCDs were microporous materials containing mesoporous structures. Because micropores give adsorption sites and mesopores supply diffusion channels, the coexistence of micropores and mesopores favor adsorption. The possible mechanism of the activation is as follows (M represent the metal oxide in the base like Na in NaOH or K in KOH) [21,22,23,24]:

$$4\mathrm{MOH}+C\to M_{2}O+H_{2}O+C\to {\mathrm{CO}}_{2}+M_{2}O\to M_{2}{\mathrm{CO}}_{3}$$

when MB was used as the activator, it was ionized into M^+^ and OH^−^ during high temperature pyrolysis. The reaction of MB with the precursor of the carbonized sample produced by-products such as carbon monoxide and hydrogen. While the hydrogen underwent chemical reactions to form water, the carbon monoxide reacted with water to form carbon dioxide. The carbon dioxide further reacted with the oxides generated by the MB to form carbonates that etched the carbonized sample. Finally, after washing with dilute acid and deionized water, the carbonates were removed to form microporous or mesoporous structures in the porous carbons.

**Figure 4:**
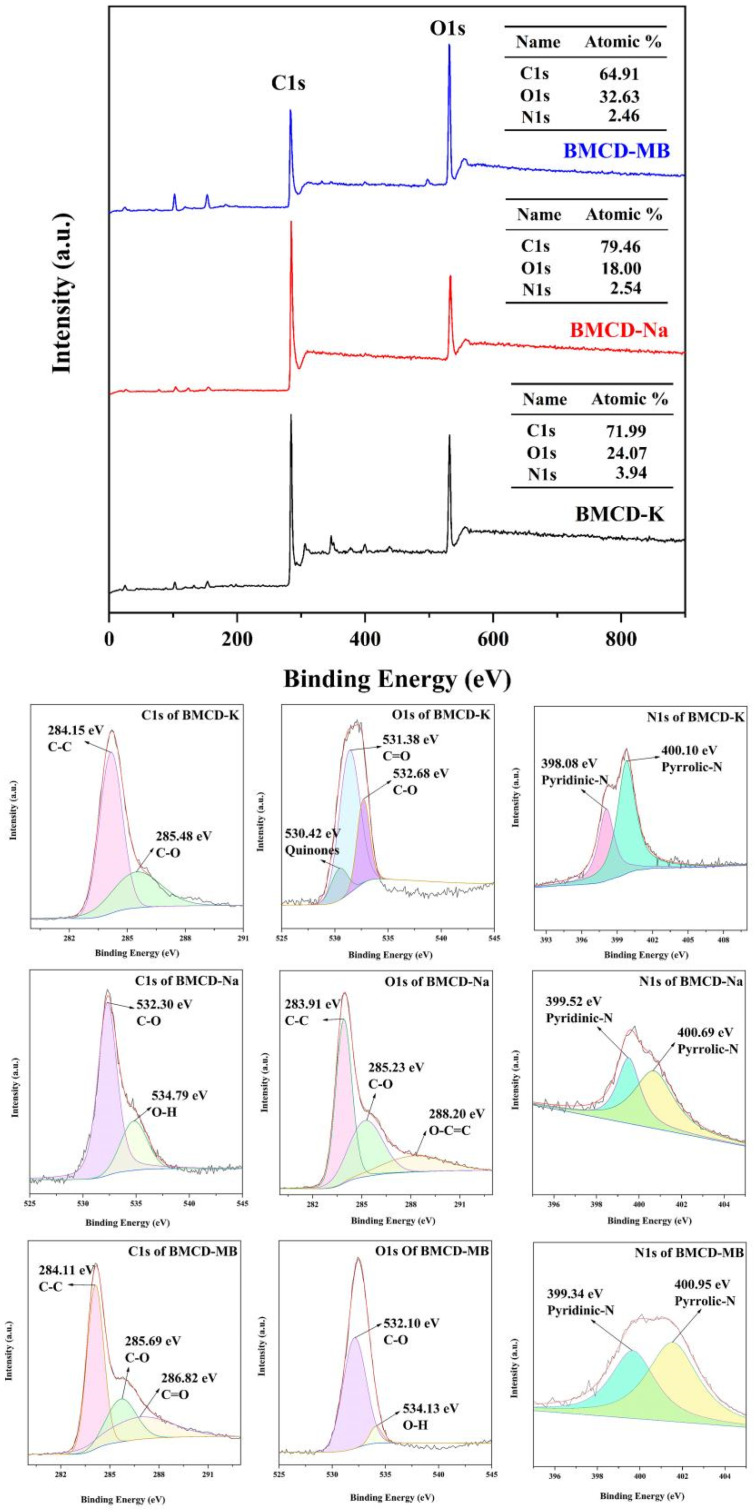
XPS spectra and the C1s, O1s, N1s of BMCD-K, BMCD-Na, and BMCD-MB.

XPS was performed to examine the surface chemistry and electronic state of the molecular of BMCDs. Like many biochars [32,33,34], BMCDs also contain mainly C, O, and N elements (Figure 4). The high-resolution C1s spectrum of the BMCDs showed four peaks, with the typical peaks at 283.91–284.15 eV corresponding to C-C, 285.23–285.69 eV corresponding to C-O, 286.82 eV corresponding to C=O, and 288.20 eV corresponding to O-C=C [25,26]. The high-resolution O1s spectrum of the BMCDs showed three peaks, with the typical peaks at 530.42 eV corresponding to quinones, 531.38 eV corresponding to C=O, 532.10–532.68 eV corresponding to C-O, and 534.13–534.79 eV corresponding to O-H [25,26]. The high-resolution N1s spectrum of the BMCDs showed two peaks, with the typical peaks at 398.08–399.52 eV corresponding to pyridinic-N and 400.10–400.95 eV corresponding to pyrrolic-N [33]. Moreover, interestingly, these functional groups are not significantly different despite the differences, which indicates that MB and its constituent single base do not significantly change their functional groups during activation. Besides, the above results, like FT-IR test results, once again indicate that the surface of BMCDs contains a large number of rich functional groups, which are likely to form hydrogen bonds with pollutant molecules during the adsorption process, thus enhancing the adsorption effect.

**Figure 5:**
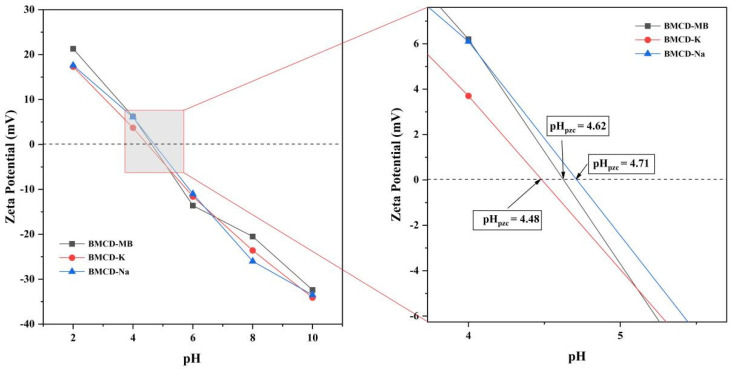
Zeta potential tests of BMCD-K, BMCD-Na, and BMCD-MB.

Zeta potential test was used to evaluate the surface charges of BMCDs, and the results were shown as Figure 5. When pH changes from 2 to 10, the surface charges of BMCDs changes from positive (21.3, 17.3, and 17.6 for BMCD-MB, BMCD-K, and BMCD-Na) to negative (−32.4, −34.1, and −33.5 for BMCD-MB, BMCD-K, and BMCD-Na). Variable surface and constant charges determine the point at which the charge is zero, often referred to as the proton interaction charge density, the constant charge density, the charge density of the inner coordination complex, and the corresponding pH (pH_pzc_), when the sum of the charge densities of the outer coordination complex is zero [35]. When the pH value is greater than pH_pzc_, the sample surface is negatively charged, and vice versa. The pH_pzc_ of all samples was less than the neutral value (7.00), indicating that BMCDs are more suitable for the treatment of cationic organic pollutants at higher pH [31]. Furthermore, the pH_pzc_ value of BMCD-MB is 4.62, which is between 4.48 of BMCD-K and 4.71 of BMCD-Na, indicating that different activators may have an effect on the preparation of biochars.

### 2.2. Adsorption Performances

#### 2.2.1. Adsorption Kinetic

**Figure 6:**
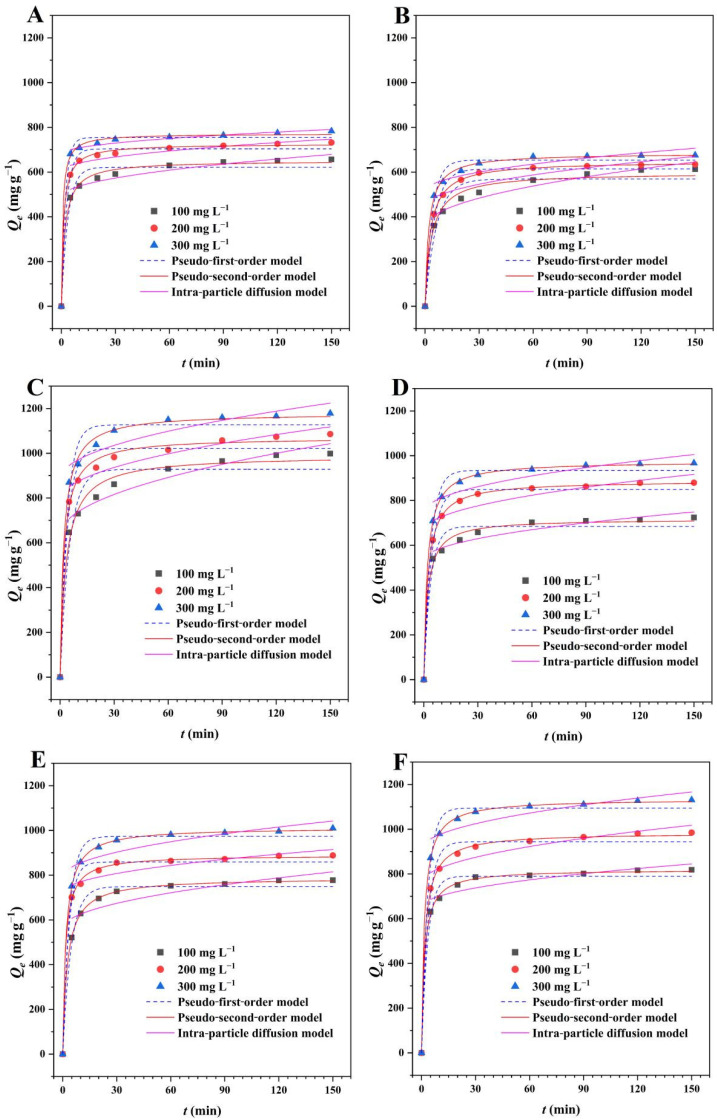
The adsorption kinetic plots of (**A**) BMCD-K, (**B**) BMCD-Na, and (**C**) BMCD-MB for RhB. The adsorption kinetic plots of (**D**) BMCD-K, (**E**) BMCD-Na, and (**F**) BMCD-MB for TH.

The adsorption kinetics describes the change of adsorbent with contact time, and is often used to study the influence of adsorbate concentration and the control mechanism of chemical reactions in the adsorption process [36,37,38]. The consequences of contact time on removal of RhB and TH (100, 200, and 300 mg L^−1^) were shown as Figure 6A–C and Figure 6D–F by BMCD-K, BMCD-Na, and BMCD-MB. From these results, it can easily be found that no matter whether RhB or TH are adsorbed, all the adsorption curves have the same trend, that is, the adsorption capacity increases sharply in the first 5 min and gradually reaches the adsorption equilibrium in 60 min, which indicates that BMCDs have a rapid adsorption rate for removal of organic pollutants. In addition, the adsorption capacity increased significantly with the increase of adsorbate concentration, indicating that the higher solution concentration, the greater the adsorption capacity to some extent.

Herein, three adsorption kinetics models including the Pseudo-first-order kinetic, Pseudo-second-order kinetic, and the Intra-particle diffusion model were investigated, and the fitting parameters were shown in Table 2. The correlation coefficients (*R*^2^) of the Pseudo-first-order kinetic were 0.9764–0.9930, 0.9523–0.9876, and 0.9559–0.9784 for BMCD-K, BMCD-K, and BMCD-MB while the adsorbate was RhB, 0.9703–0.9892, 0.9883–0.9907, and 0.9859–0.9913 while the adsorbate was TH, meanwhile, all the theoretical *Q_e.cat_* calculated according to the Pseudo-first-order kinetic model were lower than the *Q_e_* obtained from the experiments, which indicates that the Lagergren’s model probably did not play a significant role during the adsorption process [36]. While the adsorption data of RhB and TH were fitted to the Intra-particle diffusion model, the correlation coefficients (*R*^2^) were 0.8205–0.9010 and 0.7407–0.8794 for BMCD-K, 0.7264–0.9135 and 0.7349–0.7626 for BMCD-Na, and 0.8164–0.9148 and 0.7467–0.8061 for BMCD-MB, meant that the Weber-Morris’s model was not able to explain this adsorption process [37]. The Pseudo-second-order kinetic model was fitted to the adsorption data of BMCD-K, BMCD-NA, and BMCD-MB, and the correlation coefficients (*R*^2^) were 0.9956–0.9988, 0.9963–0.9998, and 0.9904-0.9971 for RhB, and 0.9935–0.9998, 0.9990–0.9998, and 0.9989–0.9998 for TH, respectively. In addition, the theoretical *Q_e.cat_* calculated according to the model were suitable with the experimental *Q_e_*, demonstrated the applicability and potential dominance of Ho-McKay’s model in the adsorption process [38]. Based on the above data, we can say that in the adsorption process of RhB and TH by BMCDs, the adsorption rate may be controlled by chemical reactions, and the similar reaction between adsorbent and adsorbate through transfer, exchange or sharing to form chemisorption bonds may promote the adsorption process.

#### 2.2.2. Adsorption Isotherm

**Figure 7:**
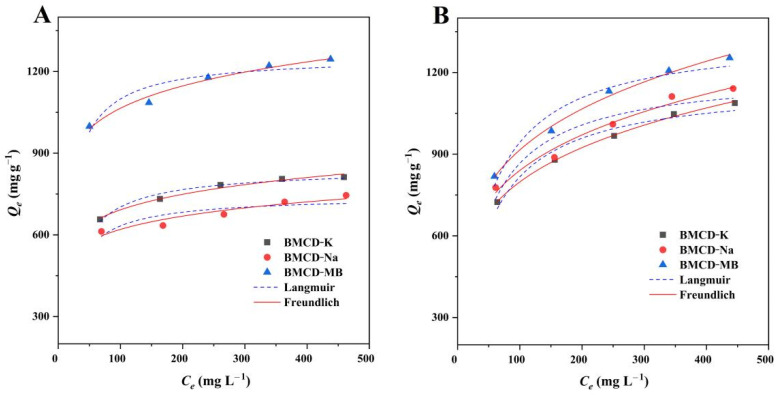
The adsorption isotherm plots of BMCD-K, BMCD-Na, and BMCD-MB for (**A**) RhB and (**B**) TH at 303 K.

Adsorption isotherms are often used to study the effect of concentration on the adsorption capacity of an adsorbent and to illustrate how the adsorbent interacts with the adsorbate during adsorption [39,40,41]. The adsorption of different initiating concentrations of RhB and TH on BMCDs were investigated at 303 K (Figure 7) and the data were fitted using Langmuir and Freundlich isotherm models (Table 3). As the concentrations of RhB and TH increase, the adsorption capacities of BMCDs increased.

The correlation coefficients (*R*^2^) were utilized to evaluate the experimental data and the results of the fit of the model equations. For RhB, the Langmuir correlation coefficients (*R*^2^) were 0.9711, 0.7327, and 0.8807 for BMCD-K, BMCD-Na, and BMCD-MB; meanwhile, the Freundlich correlation coefficients (*R*^2^) were 0.9854, 0.9823, and 0.9819. For TH, the Langmuir correlation coefficients (*R*^2^) were 0.9595, 0.8885, and 0.9485 for BMCD-K, BMCD-Na, and BMCD-MB, simultaneously, the Freundlich correlation coefficients (*R*^2^) were 0.9984, 0.9772, and 0.9933. In addition, it appears that the Freundlich isotherm model is more suitable for describing the adsorption process than the Langmuir isotherm model, suggesting that the adsorption process may not be homogeneous monolayer adsorption [39]. Furthermore, the intensity factors *n_F_* of BMCD-K, BMCD-Na, and BMCD-MB related to the adsorption intensity or surface uniformity were 9.19, 8.77, and 9.36 for RhB, 4.74, 4.81, and 4.56 for TH, which indicates that the adsorption process may involve multilayer adsorption on heterogeneous surfaces [39,40,41].

#### 2.2.3. Adsorption Thermodynamic

**Figure 8:**
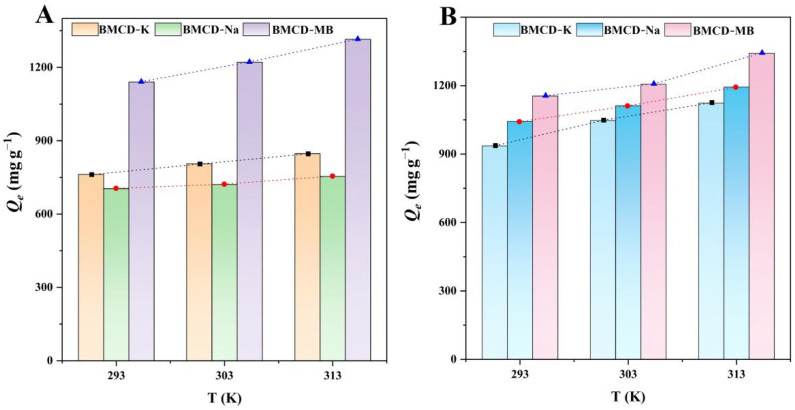
The adsorption thermodynamics of BMCD-K, BMCD-Na, and BMCD-MB for (**A**) RhB and (**B**) TH.

Distinct temperatures (293, 303, and 313 K) had different effects on the adsorption capacities of the BMCDs on RhB and TH, the results were shown as Figure 8 and the parameters were shown in Table 4. The adsorption capacities of BMCD-K, BMCD-Na, and BMCD-MB for RhB were 762, 704, and 1140 mg g^−1^, and 936, 1043, and 1154 for TH, respectively, at 293 K. As the temperature increased to 303 K, the adsorption capacities of BMCDs for RhB increased and that of TH followed the same pattern. When the temperature continues to increase further to 313 K, the increasing trend of adsorption capacities continues. Obviously, the increase of temperature promotes the adsorption process, i.e., a high-temperature environment is conducive to the adsorption of organic pollutants (RhB and TH) by BMCDs.

The Δ*G* values were all negative, suggesting that the adsorption happened spontaneously [25,26]. The standard enthalpy Δ*H* of BMCD-K, BMCD-Na, and BMCD-MB were counted as positive values (4.49, 2.87, and 6.41 kJ mol^−1^ for RhB, 8.02, 6.02, and 6.81 kJ mol^−1^ for TH), which further confirms the endothermic property ascribed to the adsorption process [25,26]. Moreover, the positive values of Δ*S* (21.51, 15.27, and 31.86 J mol^−1^ K^−1^ for RhB, 35.48, 29.67, and 33.35 J mol^−1^ K^−1^ for TH) showed an increase in the randomness of the interface between porous carbon and solution with increasing temperature [25,26].

#### 2.2.4. The Effect of pH

**Figure 9:**
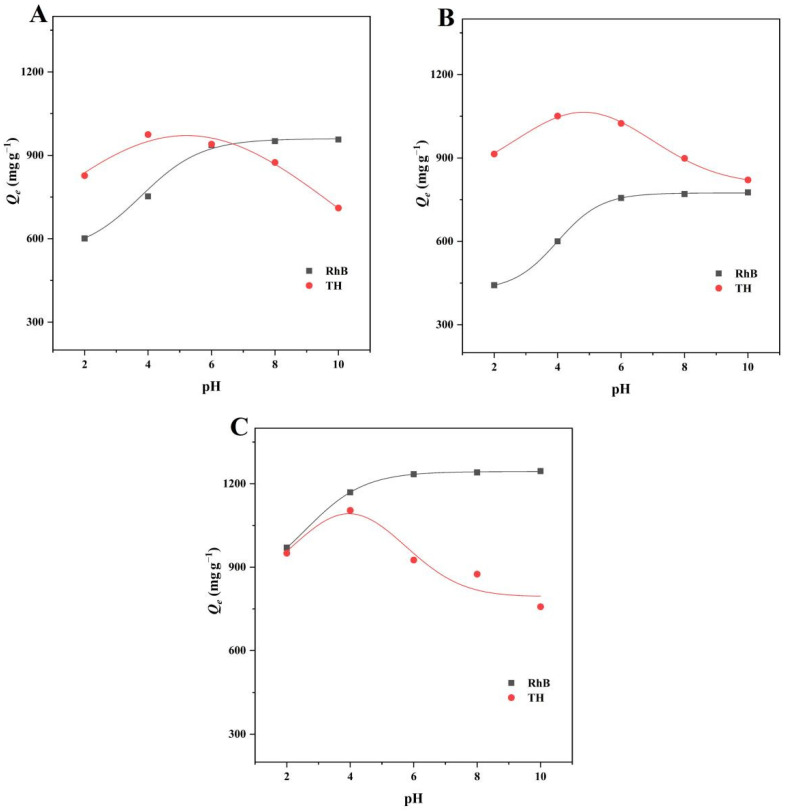
The effect of pH on the adsorption capacities of (**A**) BMCD-K, (**B**) BMCD-Na, and (**C**) BMCD-MB.

pH is an important factor which has an effect on the surface properties of the adsorbent and on the chemical properties of the solution, and can promote or inhibit the adsorption capacity of the adsorbent [42]. The effect of pH change on the adsorption capacity was investigated by varying the pH of the solution in the range of 2–10, as shown in Figure 9. The cationic dye was electrostatically attracted to the adsorbent with a surface containing more negative charges as the pH increased from 2 to 10, increasing the adsorption capacity. When the pH was more than 6, no significant increase in adsorption capacity was found. TH was found in various forms at various pH levels. When the adsorbent surface is positively charged, it is present in the cationic form (TH^+^) below pH 3.30 [43]. This might result in electrostatic repulsion and a reduction in adsorption capacity. The adsorption capacity of pH values in the range 2–6 is larger, which might be attributed to an increase in the negative charge on the adsorbent surface and a decrease in the number of positive charges in the TH solution, strengthening the adsorbent’s electrostatic attraction to TH [44]. The adsorption capacity continued to decrease as the pH increased to 10. TH was largely present in the form of anions (TH^−^ and TH^2−^) when the pH was greater than 7.68, and the substantial electrostatic repulsion with the negatively-charged adsorbent resulted in a drastic drop in the adsorption capacity [45].

### 2.3. Reusability

**Figure 10:**
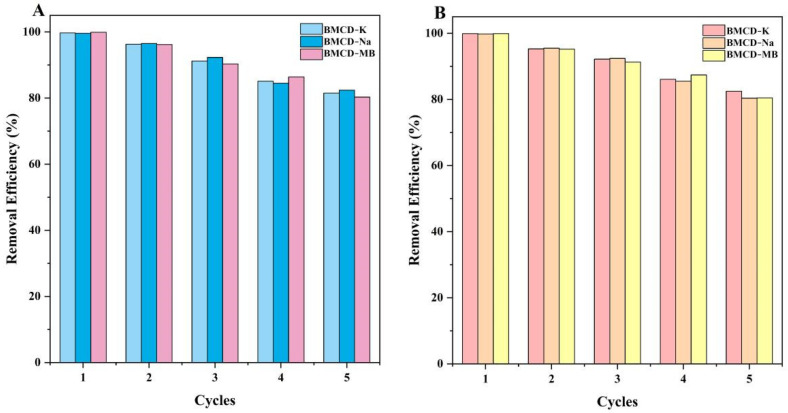
Reusability of the BMCD-K, BMCD-Na, and BMCD-MB for (**A**) RhB and (**B**) TH.

In assessing the capacity of adsorbent materials, cycling stability is an important consideration. In this investigation, five cycle experiments were used to assess the regeneration capacities of the adsorbents. Figure 10 showed the number of cycles increased, the removal efficiency and adsorption capacity of regenerated BMCDs decreased. After 5 cycles, the removal efficiency and adsorption capacity of BMCD-K, BMCD-Na, and BMCD-MB could continue to maintain 81.5%, 82.4%, and 80.3% for RhB and 82.5%, 80.4%, and 80.5% for TH compared with original BMCDs, which indicates reasonable regeneration capability and reusability.

### 2.4. The Probable Adsorption Mechanism

**Figure 11:**
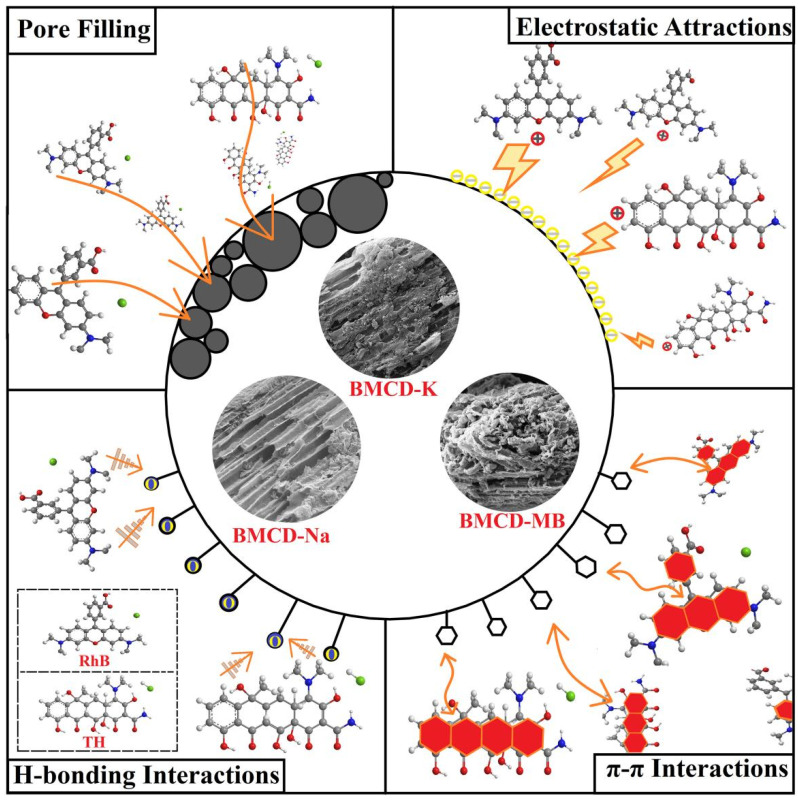
The probable adsorption mechanism diagram of BMCD-K, BMCD-Na, and BMCD-MB to RhB and TH.

In this study, the adsorption mechanisms that may affect the excellent adsorption performance of BMCDs mainly include pore filling, π-π interaction, H-bond interaction, and electrostatic attraction (Figure 11). The large surface areas (1784.6, 1446.1, and 4081.1 m^2^ g^−1^) and high pore volumes (1.1142, 1.0788, and 3.0118 cm^3^ g^−1^) of BMCD-K, BMCD-Na, and BMCD-MB provide a large number of adsorption sites, besides, the mean pore width (2.50, 2.98, and 2.95 nm) of BMCDs were larger than that of the molecular size of RhB (1.2 nm) and TH (1.3 nm), so it can be speculated that pore filling may play a dominant role in the adsorption processes. Strong electrostatic interaction exists between the organic pollutants and BMCDs with a negative zeta potential, resulting in greater adsorption capacity at higher pH aqueous environment. The presence of various functional groups on these adsorption sites that are beneficial for adsorption, such as C=O groups that may form H-bonds with the C-H groups in organic pollutants, can be seen using FT-IR spectrum data and XPS analysis findings. The results of FT-IR spectroscopy and Raman tests reveal the existence of aromatic rings in the BMCDs, which might result in π-π interactions with the aromatic rings in organic pollutants, enhancing adsorption capacities.

Besides, in the solutions of 400 mg L^−1^ at 303 K, the adsorption capacities of BMCD-K, BMCD-Na, and BMCD-MB were 951, 770, and 1241 mg g^−1^ for RhB, and 975, 1051, and 1105 mg g^−1^ for TH, respectively, which are significantly better than for most other adsorbents (Tables S1 and S2), indicating that BMCDs have great potential for removal of organic pollutants from aqueous media.

## 3. Materials and Methods

### 3.1. Materials and Reagents

Cow dung (CD) was provided by the cattle farm of the Jilin Academy of Agricultural Sciences.

HCl, KOH, and NaOH were supplied by Beijing Chemical Works (Beijing, China). Tetracycline hydrochloride (TH) and Rhodamine B (RhB) were supplied by Aladdin Chemical (Shanghai) Co., Ltd. (Shanghai, China) and the structural formulas of RhB and TH were shown as Figure S2 (Supporting Information). Mixed base (MB) was a mixture of equal mass NaOH and KOH. All the above reagents were of analytical purity grade and did not require further purification. The water involved in the rinsing and preparation process was deionized water.

### 3.2. Preparation of Cow Dung-Based Biochar Materials

The CD after carbonization is referred to as CDC. After carbonization, 2 g of activators (KOH, NaOH and MB) were used to grind 0.5 g of CDC adequately. The combined samples were heated to 700 °C at 10 °C min^−1^ in a horizontal tube furnace and kept for 90 min under nitrogen atmosphere. The activated samples were then rinsed with hydrochloric acid and ultrapure water until they had pH = 7. Samples were collected and dried at 120 °C for 12 h, then stored in desiccators. The CD-based biochar materials (BMCDs) activated with KOH, NaOH and MB were referred to as BMCD-K, BMCD-Na, and BMCD-MB.

### 3.3. Adsorption Experiments

The adsorption properties of the samples were studied using RhB and TH as organic pollutants. In equilibrium adsorption experiments, 10 mg of BMCDs was added to a flask containing 200 mL solutions. The flask was placed in a constant temperature shaker with speed of 150 rpm, and the suspension was subsequently centrifuged. After that, 1 mL of supernatant was taken and diluted with 99 mL deionized water. The concentration of the solution was determined by UV-vis spectrophotometer (Agilent Cary300, Stockton, CA, USA) at selected wavelengths (554 nm for RhB and 357 nm for TH). The standard curves of RhB and TH were shown in Figure S3. The adsorption capacities of the samples were calculated by Equation (1):

(1) $$Q_{e}=\frac{\left(C_{0}-C_{e}\right)\times V}{m}$$

where *Q_e_* (mg g^−1^) is the adsorption capacity of porous carbon, *C_e_* and *C*_0_ (mg L^−1^) denote the equilibrium and initial concentrations of the dye solution. *m* (g) and *V* (L) denote the mass of the adsorbent and the volume of the solution, respectively.

#### 3.3.1. Adsorption Kinetic Experiments

The contaminant solutions were prepared at different concentrations (100, 200, and 300 mg L^−1^). A quantity of 10 mg of BMCDs was dispersed into flasks containing 200 mL solutions and shaken at 150 rpm on a constant temperature shaker. Then the concentrations of the solutions were determined at preset time intervals.

The adsorption kinetic models including the Pseudo-first-order kinetic Equation (2), Pseudo-second-order kinetic Equation (3), and the Intra-particle diffusion model Equation (4) were used to calculate the adsorption rate constants. The models are expressed below:

(2) $$Q_{t}=Q_{e}\left(1-e^{-k_{1}t}\right)$$

(3) $$Q_{t}=\frac{k_{2}Q_{e}^{2}}{\left(1+k_{2}Q_{e}t\right)}t$$

(4) $$Q_{t}=k_{3}t^{0.5}+C$$

where *k*_1_, *k*_2_, and *k*_3_ represent the rate constants of the kinetic models, respectively, *Q_t_* is the adsorption capacity of a sample at different time points, and *C* represents the constant of the boundary layer thickness.

#### 3.3.2. Adsorption Isotherm Experiments

The contaminant solutions at different initial concentrations (100, 200, 300, 400, and 500 mg L^−1^) were prepared and used to test the adsorption isotherm at 303 K. The absorbances of the solutions were measured after adsorption saturation. The adsorption isotherm models including Langmuir Equation (5) and Freundlich Equation (6) isotherm models were used to investigate the possible adsorption mechanisms. The isotherm models are expressed below:

(5) $$Q_{e}=\frac{Q_{m}K_{L}C_{e}}{1+K_{L}C_{e}}$$

(6) Qe=KFCe1nF

where *Q_m_* (mg g^−1^) represents the maximum adsorption capacity of a sample, *C_e_* (mg L^−1^) is the solution concentration at equilibrium, *K_L_* and *K_F_* represent the constants of the Langmuir and Freundlich models, respectively. *n_F_* represent the constants of the Freundlich isotherm models.

#### 3.3.3. Adsorption Thermodynamic Experiments

The effect of temperature on the adsorption capacity of the adsorbate was investigated at an initial concentration of 400 mg L^−1^. The thermodynamic parameters standard entropy (Δ*S*), standard free Gibbs energy (Δ*G*), and standard enthalpy (Δ*H*) were analyzed to describe the effect of temperature on the adsorption process. The relationship between Δ*H*, Δ*S*, and Δ*G* could be calculated by using the following Equations (7) and (8):

(7) $$ln\left(\frac{Q_{e}}{C_{e}}\right)=\frac{\Delta S}{R}-\frac{\Delta H}{RT}$$

(8) ΔG=ΔH−TΔS

where *T* is the temperature (K), and *R* represents the gas constant (8.314 JK^−1^ mol).

#### 3.3.4. The Effect of pH on Adsorption Capacities

The variation of the adsorption capacity of the samples with pH (2, 4, 6, 8, and 10) was also investigated. The solutions were adjusted to different pH values by HCl and NaOH. After adsorption saturations were achieved, the adsorption capacities of the samples were calculated.

### 3.4. Reusability Studies

The economic efficiencies of the adsorbents were assessed by their cycling performances. A quantity of 0.1 g of BMCDs was placed into 100 mL of contaminants in each cycle (200 mg L^−1^). After adsorption, the recycled carbon was centrifuged. Following this, the BMCDs were washed with water, dried, and carbonized for 60 min at 500 °C under N_2_ protection. In the next cycle, these samples were employed as fresh adsorbents.

The details of characterization methods were shown as S1 in Supporting Information.

## 4. Conclusions

In this work, CD was used as raw material, and NaOH, KOH, and MB were used as activators to prepare biochar materials. This work not only confirmed the different effects of NaOH and KOH in the activation process, but also verified the higher efficiency of MB and prepared CD-based biochar materials with higher performance. The specific surface area and the total pore volume of BMCD-MB (4081.1 m^2^ g^−1^ and 3.0118 cm^3^ g^−1^) are significantly larger than those of BMCD-K (1784.6 m^2^ g^−1^ and 1.1142 cm^3^ g^−1^) and BMCD-Na (1446.1 m^2^ g^−1^ and 1.0788 cm^3^ g^−1^). While the synthetic dye RhB and antibiotic TH were used as organic pollutants models, the maximum adsorption capacities of BMCD-K, BMCD-Na, and BMCD-MB were 951, 770, and 1241 mg g^−1^ for RhB, and 975, 1051, and 1105 mg g^−1^ for TH, respectively. Furthermore, the adsorption capacities of BMCDs to RhB and TH can still be over 80% after 5 cycles. This work not only prepared a series of biochar materials (BMCDs) with enhanced performance, which can better cope with organic pollutants wastewater treatment, but more importantly, it provided a new strategy for the exploration of other biomasses. In the future, we will continue to explore the types and uses of MB in depth to further explore the activation potential of MB.

## Figures and Tables

**Table 1:** The data of N_2_ adsorption-desorption for BMCDs prepared via different activators.

Samples	S_BET_ (m^2^ g^−1^)	V_micro_ (cm^3^ g^−1^)	P_m_ (nm)	V_total_ (cm^3^ g^−1^)
CD	0.9	-	31.93	0.0071
CDC	23.8	-	7.34	0.0436
BMCD-K	1784.6	1.0239	2.50	1.1142
BMCD-Na	1446.1	0.9124	2.98	1.0788
BMCD-MB	4081.1	2.5583	2.95	3.0118

S_BET_ (m^2^ g^−1^) is the BET specific surface area, V_micro_ (cm^3^ g^−1^) is the volume of micropores, P_m_ (nm) is the mean pore size, and V_total_ (cm^3^ g^−1^) is the total pore volume.

**Table 2:** Fitting parameters of adsorption kinetic models for RhB and TH at 303 K.

Organic Pollutants	Samples	Kinetic Models	Parameters	Concentration *C*_0_ (mg L^−1^)		
				100	200	300
RhB	BMCD-K		*Q_e_* (mg g^−1^)	657	732	783
		Pseudo-first-order	*k*_1_ (min^−1^)	0.2704	0.3414	0.4440
			*Q_e.cat_* (mg g^−1^)	622	704	754
			*R* ^2^	0.9764	0.9914	0.9930
		Pseudo-second-order	*k*_2_ (g mg^−1^ min^−1^)	0.0007	0.0011	0.0017
			*Q_e.cat_* (mg g^−1^)	661	736	791
			*R* ^2^	0.9956	0.9988	0.9985
		Intra-particle diffusion	*k_3_* (mg g^−1^ min^−0.5^)	15.57	11.78	8.99
			*C*	488	586	680
			*R* ^2^	0.8857	0.8205	0.9010
	BMCD-Na		*Q_e_* (mg g^−1^)	613	634	676
		Pseudo-first-order	*k*_1_ (min^−1^)	0.1556	0.1948	0.2493
			*Q_e.cat_* (mg g^−1^)	569	614	654
			*R* ^2^	0.9523	0.9876	0.9836
		Pseudo-second-order	*k*_2_ (g mg^−1^ min^−1^)	0.0004	0.0005	0.0007
			*Q_e.cat_* (mg g^−1^)	617	648	684
			*R* ^2^	0.9463	0.9998	0.9987
		Intra-particle diffusion	*k*_3_ (mg g^−1^ min^−0.5^)	23.87	18.33	15.79
			*C*	353	445	513
			*R* ^2^	0.9135	0.7264	0.7652
	BMCD-MB		*Q_e_* (mg g^−1^)	998	1086	1178
		Pseudo-first-order	*k*_1_ (min^−1^)	0.1916	0.2521	0.2593
			*Q_e.cat_* (mg g^−1^)	928	1021	1127
			*R* ^2^	0.9559	0.9761	0.9784
		Pseudo-second-order	*k*_2_ (g mg^−1^ min^−1^)	0.0003	0.0004	0.0005
			*Q_e.cat_* (mg g^−1^)	999	1091	1180
			*R* ^2^	0.9904	0.9956	0.9971
		Intra-particle diffusion	*k*_3_ (mg g^−1^ min^−0.5^)	33.53	26.83	23.88
			*C*	632	790	882
			*R* ^2^	0.9148	0.8863	0.8164
TH	BMCD-K		*Q_e_* (mg g^−1^)	724	879	967
		Pseudo-first-order	*k*_1_ (min^−1^)	0.2659	0.2399	0.2354
			*Q_e.cat_* (mg g^−1^)	684	849	935
			*R* ^2^	0.9703	0.9891	0.9892
		Pseudo-second-order	*k*_2_ (g mg^−1^ min^−1^)	0.0006	0.0005	0.0005
			*Q_e.cat_* (mg g^−1^)	727	888	975
			*R^2^*	0.9935	0.9998	0.9998
		Intra-particle diffusion	*k*_3_ (mg g^−1^ min^−0.5^)	17.63	20.77	21.20
			*C*	533	662	746
			*R* ^2^	0.8794	0.7407	0.7497
	BMCD-Na		*Q_e_* (mg g^−1^)	778	888	1010
		Pseudo-first-order	*k*_1_ (min^−1^)	0.2138	0.3077	0.2705
			*Q_e.cat_* (mg g^−1^)	749	859	973
			*R* ^2^	0.9883	0.9889	0.9907
		Pseudo-second-order	*k*_2_ (g mg^−1^ min^−1^)	0.0005	0.0007	0.0005
			*Q_e.cat_* (mg g^−1^)	788	890	1013
			*R* ^2^	0.9998	0.9990	0.9998
		Intra-particle diffusion	*k*_3_ (mg g^−1^ min^−0.5^)	20.77	15.83	15.52
			*C*	560	720	791
			*R* ^2^	0.7404	0.7626	0.7349
	BMCD-MB		*Q_e_* (mg g^−1^)	818	985	1131
		Pseudo-first-order	*k*_1_ (min^−1^)	0.2886	0.2721	0.2940
			*Q_e.cat_* (mg g^−1^)	790	944	1095
			*R* ^2^	0.9877	0.9859	0.9913
		Pseudo-second-order	*k*_2_ (g mg^−1^ min^−1^)	0.0007	0.0006	0.0006
			*Q_e.cat_* (mg g^−1^)	820	985	1135
			*R* ^2^	0.9989	0.9990	0.9998
		Intra-particle diffusion	*k*_3_ (mg g^−1^ min^−0.5^)	15.83	21.17	20.94
			*C*	651	758	910
			*R* ^2^	0.7626	0.8061	0.7467

**Table 3:** Fitting parameters of adsorption isotherm models for RhB and TH at 303 K.

Organic Pollutants	Samples	Isotherm Types	Parameters	
RhB	BMCD-K	Langmuir	*Q_m_* (mg g^−1^)	843
			*K_L_* (L mg^−1^)	0.0501
			*R* ^2^	0.9711
		Freundlich	*K_F_* (mg g^−1^(L mg^−1^)^1/n^)	376
			*n_F_*	9.19
			*R* ^2^	0.9854
	BMCD-Na	Langmuir	*Q_m_* (mg g^−1^)	742
			*K_L_* (L mg^−1^)	0.0580
			*R* ^2^	0.7327
		Freundlich	*K_F_* (mg g^−1^(L mg^−1^)^1/n^)	410
			*n_F_*	8.77
			*R* ^2^	0.9823
	BMCD-MB	Langmuir	*Q_m_* (mg g^−1^)	1256
			*K_L_* (L mg^−1^)	0.0703
			*R* ^2^	0.8807
		Freundlich	*K_F_* (mg g^−1^(L mg^−1^)^1/n^)	651
			*n_F_*	9.36
			*R* ^2^	0.9819
TH	BMCD-K	Langmuir	*Q_m_* (mg g^−1^)	1161
			*K_L_* (L mg^−1^)	0.0238
			*R* ^2^	0.9595
		Freundlich	*K_F_* (mg g^−1^(L mg^−1^)^1/n^)	302
			*n_F_*	4.74
			*R* ^2^	0.9984
	BMCD-Na	Langmuir	*Q_m_* (mg g^−1^)	1203
			*K_L_* (L mg^−1^)	0.0258
			*R* ^2^	0.8885
		Freundlich	*K_F_* (mg g-1(L mg^−1^)^1/n^)	323
			*n_F_*	4.81
			*R* ^2^	0.9772
	BMCD-MB	Langmuir	*Q_m_* (mg g^−1^)	1341
			*K_L_* (L mg^−1^)	0.0237
			*R* ^2^	0.9485
		Freundlich	*K_F_* (mg g^−1^(L mg^−1^)^1/n^)	333
			*n_F_*	4.56
			*R* ^2^	0.9933

**Table 4:** Fitting adsorption thermodynamic parameters for RhB and TH.

Organic Pollutants	Samples	T (K)	∆*G* (kJ mol^−1^)	∆*H* (kJ mol^−1^)	∆*S* (J mol^−1^ K^−1^)
RhB	BMCD-K	293	−1.81	4.49	21.51
		303	−2.03		
		313	−2.24		
	BMCD-Na	293	−1.60	2.87	15.27
		303	−1.72		
		313	−1.91		
	BMCD-MB	293	−2.93	6.41	31.86
		303	−3.23		
		313	−3.56		
TH	BMCD-K	293	−2.37	7.98	35.48
		303	−2.78		
		313	−3.08		
	BMCD-Na	293	−2.67	6.02	29.67
		303	−2.95		
		313	−3.27		
	BMCD-MB	293	−2.96	6.81	33.35
		303	−3.19		
		313	−3.63		

## Data Availability

The study did not report any data.

## Supplementary Materials

The supporting information can be downloaded at: https://www.mdpi.com/article/10.3390/ijms231710094/s1. References [22,46,47,48,49,50,51,52,53,54,55,56,57,58,59,60,61,62,63,64,65,66,67,68,69,70,71,72,73,74] are cited in the supplementary materials.
