# Supplementary material for: Cow Dung-Based Biochar Materials Prepared via Mixed Base and Its Application in the Removal of Organic Pollutants
Source: Int J Mol Sci. 2022 Sep 3;23(17):10094. doi: 10.3390/ijms231710094 (PMC9456264; doi:10.3390/ijms231710094)
Supplement: Supplementary file 1 [file ijms-23-10094-s001.zip › ijms-1895317-supplementary.pdf]

Cow Dung-Based Biochar Materials Prepared via Mixed Base and Its Application in the Removal of Organic Pollutants

Xiaoxin Chen[1,2], Gengxin Yu[1,2], Yuanhui Chen[2], Shanshan Tang[1,2], Yingjie Su[1,2,*]

[1.]College of Life Sciences, Jilin Agricultural University, Changchun 130118, China

[2.]Key Laboratory of Straw Comprehensive Utilization and Black Soil Conservation, Ministry of Education, Jilin Agricultural University, Changchun 130118, China

*Corresponding author E-mail: yjsu@jlau.edu.cn

S1. Characterization methods

Thermogravimetric analysis of the samples was carried out under the protection of nitrogen flow (TGA, Netzsch STA409PC, Germany). Scanning electron microscopy was used to examine the morphology of materials (SEM, ZEISS SIGMA HD, Germany). FT-IR spectrometer was used to characterize the surface functional groups of materials between 400 and 4000 cm^{-1} at a resolution of 1 cm^{-1} (FT-IR, Thermo Fisher Nicolet iS50, USA). X-ray diffraction patterns of the powders were observed by an X-ray diffractometer with a filtered Cu-Ka X-ray source (XRD, Bruker D8 Advance, Germany). Raman spectra of the samples were obtained using a model Renishaw 2000 Raman spectrometer at 514 nm to investigate the presence of defects in the biochar materials. N_2 adsorption-desorption isotherms were used to obtain the porosity of the samples at 77 K (N_2 adsorption-desorption isotherms, Quantachrome Autosorb iQ2, USA). The Brunauer-Emmett-Teller (BET) theory was used to calculate the surface area. The non-local density functional theory (NLDFT), HK method (HK), and the Barrett-Joyner-Halenda (BJH) model were used to analyse the pore size distribution of samples. X-ray photoelectron spectroscopy was used to test the electronic binding energy of the samples (XPS, Thermo Escalab 250Xi+, USA). The zeta potential instrument was used to characterize the surface charge of samples (Zeta potential, Zetasizer Nano ZS90, UK).

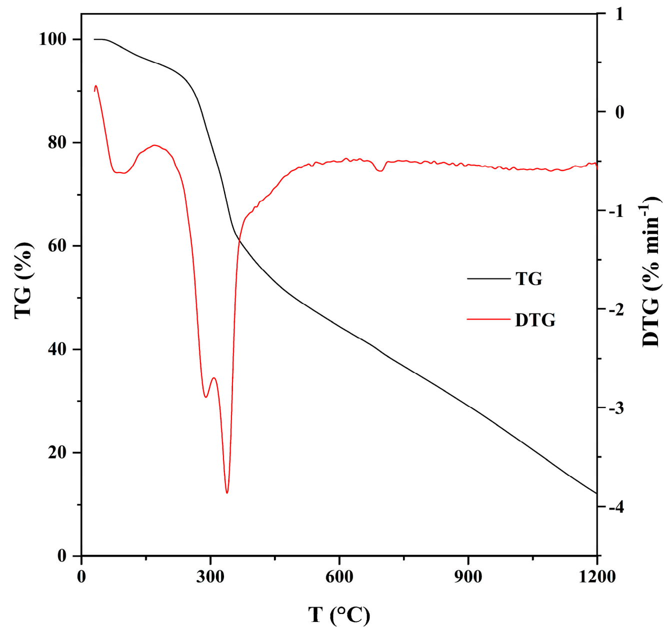

Figure S1 The TGA and DTG curves of CD.

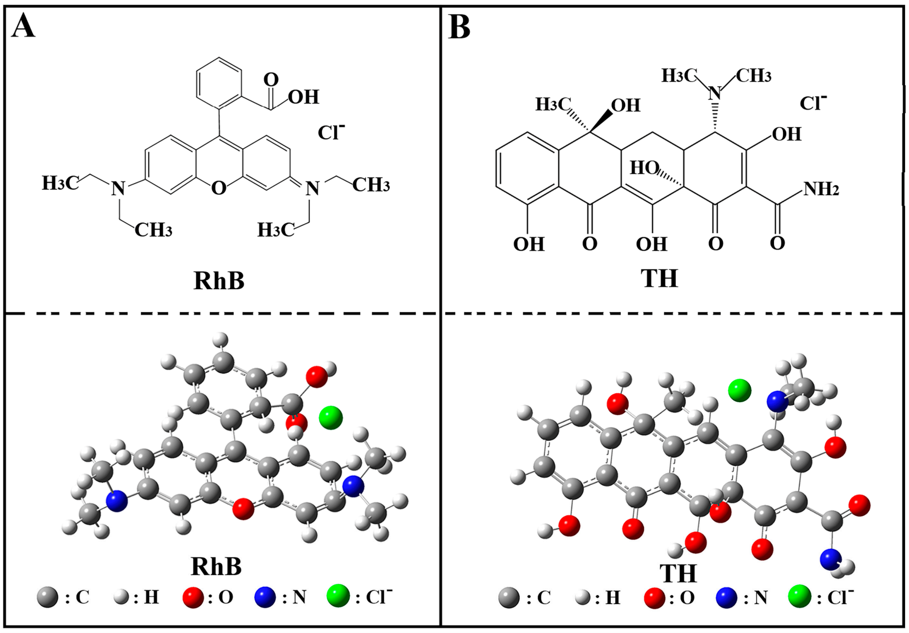

Figure S2 The structural formulas of (A) RhB and (B) TH.

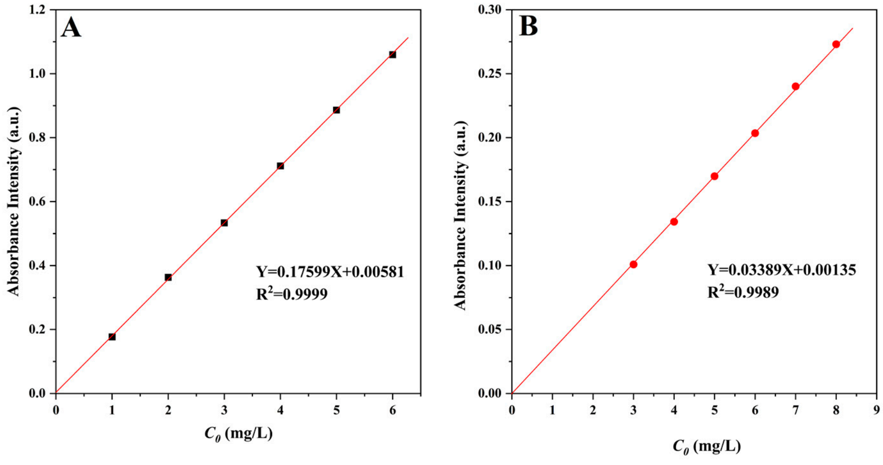

Figure S3 The standard curves of (A) RhB and (B) TH.

Table S1. Comparison of the adsorption capacities of samples to RhB with other adsorbents.

Adsorbent	Q_e (mg g^{-1})	References
Functionalized graphene via tannic acid	201	[46]
Polymer nanocomposite was prepared using formaldehyde and resorcinol	208	[47]
Tannery residual biomass (TRB)	250	[48]
Gelatin/activated carbon composite beads	256	[49]
Activated carbon prepared from bagasse pith	264	[50]
Polymer modified biomass of baker's yeast	267	[51]
Activated carbon derived from scrap tires	307	[52]
Magnetic AC/CeO_2	325	[53]
Pyruvic acid (PA)-modified activated carbons	385	[54]
N-vinylimidazole modified hyper-cross-linked resins	421	[55]
Treated rice husk-based activated carbon	518	[56]
Gum ghatti and Fe_3O_4 magnetic nanoparticles based nanocomposites	655	[57]
Raphia hookerie fruit epicarp	667	[58]
Porous carbon based quinoa husk	759	[59]
Porous carbon based on corn straw	1578	[22]
BMCD-Na	770	This work
BMCD-K	951	This work

BMCD-MB	1241	This work

Table S2 Comparison of the adsorption capacities of samples to TH with other adsorbents.

Adsorbent	Q_e (mg g^{-1})	References
Rice husk ash	8	[60]
Red earth clay composite	15	[61]
Grapefruit peel	33	[62]
Poplar saw dust	61	[63]
BCFS800	61	[64]
BM-biochars	85	[65]
Chicken bone	99	[66]
Municipal sludge	122	[67]
MGO	141	[68]
x-mAC	222	[69]
Graphene oxide	314	[70]
NaOH-activated carbon	455	[71]
Magnetic carbon-coated cobalt oxide nanoparticles	769	[72]
SG-ELBC	1163	[73]
Encapsulated phosphotungstic acid (PTA) MIL-53 (Fe)	1250	[74]
BMCD-K	975	This work
BMCD-Na	1051	This work
BMCD-MB	1105	This work
